# Effects of Trunk Exercise on Unstable Surfaces in Persons with Stroke: A Randomized Controlled Trial

**DOI:** 10.3390/ijerph17239135

**Published:** 2020-12-07

**Authors:** Pei-Yun Lee, Jhen-Cih Huang, Hui-Yu Tseng, Yi-Ching Yang, Sang-I Lin

**Affiliations:** 1 Department of Physical Therapy, College of Medicine, National Cheng Kung University, Tainan 70101, Taiwan; peiyunlee@mail.ncku.edu.tw (P.-Y.L.); ayj104x@gmail.com (J.-C.H.); 2 Department of Rehabilitation Medicine, Tainan Hospital, Ministry of Health and Welfare, Tainan 701, Taiwan; habyui@yahoo.com.tw; 3 Department of Family Medicine, National Cheng Kung University Hospital, College of Medicine, National Cheng Kung University, Tainan 70101, Taiwan; yiching@mail.ncku.edu.tw

**Keywords:** stroke, trunk performance, walking, standing balance, trunk training

## Abstract

*Background*: Improving balance-related ability is an important goal in stroke rehabilitation. Evidence is needed to demonstrate how this goal could be better achieved. *Aim*: Determine if trunk exercises on unstable surfaces would improve trunk control and balance for persons in the subacute stage of stroke. *Design*: An assessor-blind randomized controlled trial. *Setting*: Inpatients in the department of rehabilitation in a general hospital. *Population*: Patients who suffered a first-time stroke with onset from one to six months. *Methods*: Inpatients with stroke were assigned to upper limb exercises (control group, *n* = 17) or trunk exercises on unstable surfaces (experimental group, *n* = 18) to receive training twice a week for six weeks, in addition to their daily conventional stroke rehabilitation. Sensorimotor function tests, including hand grip, plantar sensitivity, stroke rehabilitation assessment of movement and Fugl-Meyer lower extremity motor scale, and clinical outcome assessments, including Trunk Impairment Scale and 6 m walk test, were conducted before and after six weeks of training. The center of the pressure area while maintaining static posture and peak displacement while leaning forward, as well as the average speed of raising the unaffected arm, were measured in sitting without foot support, sitting with foot support and standing to reflect trunk control, sitting balance and standing balance, respectively. *Results*: The between-group differences in the sensorimotor functions were nonsignificant before and after training. Compared with the control group, the experimental group had significantly greater forward leaning and faster arm raising in sitting without foot support, higher Trunk Impairment Scale total score, and shorter 6 m walking time after training, but not before training. *Conclusion*: Trunk exercises on unstable surfaces could further improve trunk control, the ability to raise the unaffected arm rapidly in sitting, and walking for persons in the subacute stage of stroke. This intervention may be considered to be included in stroke rehabilitation.

## 1. Introduction

Stroke is a common health problem that could disrupt the central neural pathways and lead to impairments in sensory and motor function and central processing. Even after intensive rehabilitation, persons with stroke are often left with functional deficits, making stroke the leading cause of disability in adults globally [1]. Persons with stroke also have higher rates of falling than the general older populations and therefore are at a higher risk of further disability, hospitalization, and mortality [2]. Although disability and falling are multifactorial problems, impaired balance ability is a common and important underpinning factor [3,4].

Balance impairments have been reported to occur in 17–83% of persons with stroke [5,6] and can be manifested in different ways. Greater postural sway while maintaining quiet sitting or standing, as well as a smaller range in moving the body’s center of mass (COM), has been found to occur after stroke [7,8]. The ability to provide a stable foundation for the performance of upper limb tasks has also been found to be impaired and lead to upper limb movement limitations [9,10].

One of the primary factors for balance impairments after stroke is trunk control impairments. Stroke can lead to impairments in trunk muscle strength [11,12,13], activation patterns [10,11,12] and position sense [14]. These problems and their subsequent trunk performance deficits have been found to be related to imbalance and limited functional recovery [15,16]. Following this line of thinking, trunk exercises could thus possibly improve balance and functional recovery. There is evidence showing that sitting or supine lying trunk exercises, including core stability, reaching and weight shift exercises, proprioceptive neuromuscular facilitation, could improve trunk control, sitting and standing balance and mobility for person in the subacute stage (one–six months after onset) of stroke [17,18,19].

In spite of the existing information, there are still knowledge gaps limiting our understanding. In previous studies, sitting balance was assessed with the participants’ feet supported. It is thus unclear if the effects of trunk exercises were mediated by improved trunk control per se or better use of lower limbs for balance. It is also unclear if the ability to provide a stable foundation for the performance of rapid upper limb movements would be improved by trunk exercises. Filling these knowledge gaps could provide information for the planning of balance training in stroke rehabilitation.

Support surface is critical for maintaining balance by allowing the generation of ground reaction forces to counteract imbalancing forces. Studies on healthy adults showed that when balancing on unstable surfaces, continuous and larger postural perturbations would occur and require prolonged and increased activations of postural muscles [20,21,22]. What is more, because the direction of these postural perturbations would be different, different patterns of inter-muscular coordination would also be elicited when balancing on unstable surfaces [23]. It has been shown that to achieve similar effects, a lower training load could be used when unstable surfaces were used [24,25].

The higher neuromuscular control and task demands related to balancing on unstable surfaces could be particularly beneficial for persons with stroke for whom larger postural perturbations may not be safe. In addition, for those in the subacute stage of stroke, when the recovery of the affected side neuromuscular control is still likely [26], a smaller training load may facilitate motor recovery of the affected side, rather than lead to the development of compensatory movements. A recent systematic review also suggested that trunk training on unstable surfaces could have a greater effect than on stable surfaces for persons with stroke [27].

The purpose of this study was to examine the effects of trunk exercises on unstable surfaces on different domains of balance ability for persons in the subacute stage of stroke. It was hypothesized that trunk exercise training on unstable surfaces would significantly improve trunk control and balance.

## 2. Materials and Methods

### 2.1. Study Design and Setting

This study was an assessor-blinded randomized controlled trial designed to examine the effect of trunk exercises on unstable surfaces on balance ability in persons with subacute stroke. The study design was approved by the Institutional Review Board of the National Cheng Kung University Hospital (protocol number: B-ER-106-428, approval date: 1st August, 2018, chairperson: Sang-I Lin) and was registered at CliniclTrials.gov Protocol Registration and Results System (trial number NCT04434443). All the research methods were performed in accordance with the provisions of the Declaration of Helsinki (as revised in Tokyo 2004). All participants provided their written informed consents before participation in the study. The study, including assessment and training, was conducted in a multi-purpose exercise room in the hospital where the participants were recruited.

### 2.2. Participants

In-patients in the department of rehabilitation in the hospital where the study was conducted were recruited. The inclusion criteria were patients with first-time stroke with onset from one to six months, who were able to sit without support for at least 30 s and follow experimental instructions. The exclusion criteria were age over 80 or having musculoskeletal or other neuromuscular conditions that could affect balance. To minimize measurement ceiling effects, those who obtained the maximum score in the Trunk Impairment Scale (TIS, maximal score = 23) were excluded. All the participants were receiving daily physical therapy for stroke, including mobility training and muscle strengthening during the study period.

Ninety-eight persons in the subacute stage of stroke were contacted between August 2018 and October 2019. A total of 38 participants fulfilled the eligibility criteria. After completing the baseline assessment, the participants were randomly allocated into either the control (CON, *n* = 19) or experimental (EXP, *n* = 19) group. Randomization was conducted by using a computer randomization program. The randomization schedule was concealed to the assessor.

### 2.3. Sensorimotor Function and Walking Assessment

Participants went through a series of clinical sensorimotor function and walking tests. The Stroke Rehabilitation Assessment of Movement (STREAM) was used to assess overall motor function and mobility. The TIS was used to assess trunk performance. The Fugl-Meyer lower extremity motor scale (FMLE motor) was used to measure the lower limb motor function. The modified Ashworth scale was used to grade the spasticity of the lower extremity. Plantar cutaneous sensation of the big toe was measured using the Semmes-Weinstein monofilaments (Semmes-Weinstein Aesthesiometer, Bolingbrook, IL, USA). The JAMAR^®^ Hand Dynamometer (Sammons Preston, Mississauga, ON, Canada) was used to assess the grip strength of the unaffected limb to represent the overall strength of the unaffected upper limb. The 6 m walk test was used to measure walking ability. In this test, the participant walked for 8 m at their preferred speeds and the time taken to walk the 2nd~7th meter was recorded using a digital stopwatch. The testers of these clinical tests were not aware of the group assignment and had no access to the related data.

### 2.4. Balance Assessment

Three domains of sitting and standing balance ability were tested: postural stability, moving the center of mass (COM) forward, and providing a stable base for the performance of rapid upper limb motion. For postural stability, the instruction was to look ahead and maintain upright posture for 20 s. For moving the COM, the instruction was to lean forward as far as possible without losing balance. For the arm raising task, the instruction was to raise the unaffected arm to the ceiling as fast as possible. A gyroscope sensor (Trigno^®^ Avanti Sensors, Delsys Incorporated, Natick, MA, USA) was placed on the lateral side of the radial styloid process at the wrist to measure the angular velocity of the arm raising movement.

There were three testing positions: sitting with foot support, sitting without foot support, and standing. The two sitting positions were tested first in a random order, followed by the standing position. All the tasks within each testing position were tested in a random order. For sitting tests, a customized height adjustable chair with an imbedded force platform (Kistler, 9286B, Winterthur, Switzerland) was used. The participant sat without back support, with two-thirds of their thighs supported by the seat, arms relaxed, and hands on thighs. In sitting in the foot support condition, the participant sat with their feet shoulder-width apart and flat on the ground to test sitting balance. In sitting in the without-foot-support condition, the chair was elevated and the participant sat with their lower legs dangling to test the ability to use the trunk but not the lower limbs for balance, i.e., trunk control. For all the sitting tests, the outlines of the base of support (i.e., buttocks and thighs) were traced onto a sheet to be used again in the post-training assessment to ensure the consistency of the base of support. For standing tests, the participant stood on the force platform with their feet shoulder-width apart without assistive devices. The outlines of their feet were traced to be used in the post-training assessment.

### 2.5. Intervention

The participants were randomly assigned into the control (CON) or experimental (EXP) group to receive 30 min training, two non-consecutive days per week for a total of six weeks. A trained physical therapist experimenter carried out the training for both groups. The CON received upper limb range of motion exercises at comfortable speeds in a well-supported sitting position.

The EXP group received trunk exercises training in hook-lying and sitting (Table 1). There were four exercises in the hook-lying position: (1) abdominal draw-in maneuver with a balance pad (AIREX^®^, 48 × 40 × 6 cm, Sins, Switzerland) under the buttocks, (2) abdominal muscles isometric contraction, (3) lower trunk rotation, and (4) bridging combining with abdominal draw-in maneuver. For exercises 2~4, the level of support surface instability would be increased gradually by first placing the balance pad under the feet, then placing a BOSU ball (26 cm in diameter, 21.6–22.9 cm in height when inflated; BOSU^®^, Ashland, OH, USA) under the feet, and then finally placing the balance pad under the buttocks and the BOSU ball under the feet.

There were two levels of sitting exercises (Table 1). For the first level, the participant sat without back or foot support first on the balance pad, then progressed to sitting on the BOSU ball. There were five exercises in this level: (1) pelvic anterior and posterior tilt, (2) pelvic lateral tilt, (3) trunk flexion, extension and rotation, (4) affected arm lateral reach with a Swiss ball (55 cm, Theragear^®^ Inc., Mission, BC, Canada) under the arm for support and guidance, and (5) pelvic rotation. For the second level, the participant sat on a Swiss ball (65 cm) with their feet flat on the ground. There were five exercises in this level: (1) maintaining quiet sitting with chest expansion exercises, (2) pelvic anterior and posterior tilt, (3) pelvic lateral tilt, (4) stepping, and (5) stepping with arm swing.

### 2.6. Outcome Measures

The primary outcome measure was the center of pressure (COP) motion derived from the force platform. For postural stability, the variable of interest was the area of postural sway. For moving the COM forward (forward leaning), the variable of interest was the peak COP forward displacement. For the unaffected arm raising, the variable of interest was the averaged arm raising speed. It has been shown that the ability to provide a stable foundation was critical to the performance of arm raising [28,29]. Thus, a faster unaffected arm raising speed would be indicative of better trunk stability or balance. The secondary outcome measures were the total score of the Trunk Impairment Scale (TIS) and 6 m walk time. The inter-rater and intra-rater reliability for TIS were examined using the intraclass correlation and found to be 0.893 and 0.896, respectively.

### 2.7. Statistical Analysis

The Shapiro–Wilk test of normality was conducted for all the parameters. For the comparisons of the basic characteristics, independent *t* (for stroke onset duration and body height), Mann–Whitney U (for age, body weight, grip strength, FMLE motor, plantar sensitivity, and STREAM), and Chi-square (for gender and affected side) tests were used. For the primary outcome variables, those that were normally distributed and had equal variances based on Levene’s test of equality would be analyzed using repeated measures analysis of variance (ANOVA) to determine the effects of time (repeated) and group. For variables with significant interactions after ANOVA, independent *t* tests were used to compare between-group differences in pre- and post-training. GPower version 3.1 F test for ANOVA: repeated measures, within-between interactions, was used for post-hoc power analysis for the primary outcome measures. For those that were not normally distributed or had unequal variances, the Mann–Whitney U tests were used to compare the between-group differences in pre- and post-training. The significance level was set at 0.05.

The results of the Shapiro–Wilk test of normality showed that forward leaning distance and arm raising velocity in all three conditions, as well as TIS data, were normally distributed and had equal variances. These variables were analyzed using ANOVA. Sway area in all three conditions and 6 m walk time were not normally distributed and arm raising in sitting with foot support and in standing had unequal variances. These variables were analyzed using Mann–Whitney U tests.

### 2.8. Data Availability

The data associated with this study are not publicly available but are available from the corresponding author upon reasonable request.

## 3. Results

**Figure 1:**
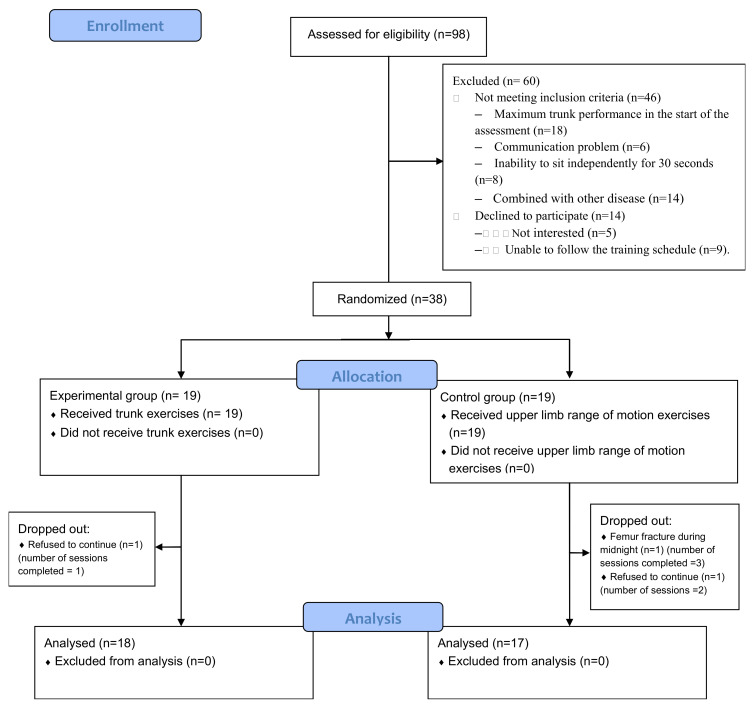
Flow chart of the study.

Two participants in CON dropped out of the study after two and three training sessions, and one participant in EXP dropped out of the study after one training session (Figure 1). Therefore, the data of 17 participants in CON and 18 participants in EXP were entered for analysis. The basic characteristics of the two groups are shown in Table 2 and the between-group differences were nonsignificant.

### 3.1. Sensorimotor Function and Walking

The two groups did not differ significantly in their unaffected side grip strength, affected side plantar sensitivity, FMLE motor or STREAM before or after training (Table 3). For TIS, there was significant time x group interaction (F(1,33) = 7.934, *p* = 0.008), and post-hoc analysis of between-group differences showed significantly higher scores in EXP in post-training but not in pre-training (Table 3). Before training, there were 11 and 17 participants who were able to complete the 6 m walk test in CON and EXP, respectively, and the between-group difference was nonsignificant. After training, all the participants were able to complete the walking test and EXP had significantly shorter walking time (Table 3).

### 3.2. Sitting Performance

While maintaining quiet sitting with foot support, the differences in sway area between the two groups were nonsignificant pre- and post-training (Table 3). For forward leaning, there was significant group (F(1,33) = 5.069, *p* = 0.031) and time (F(1,33) = 4.632, *p* = 0.039) main effect but not interaction (F(1,33) = 1.452, *p* = 0.237, power = 0.991). For unaffected arm raising speed, the differences between the two groups were nonsignificant pre- and post-training (Table 3).

While maintaining quiet sitting without foot support, the differences in sway area between the two groups were nonsignificant pre-training but were significantly greater in EXP post-training (Table 3). For forward leaning, there was significant time x group interaction (F(1,33) = 9.382, *p* = 0.004, power = 0.999), and post-hoc analysis of between-group differences showed significantly greater forward leaning distance in EXP in post-training, but not in pre-training (Table 3). For unaffected arm raising speed, there was significant time x group interaction (F(1,33) = 4.878, *p* = 0.034, power = 0.996), and post-hoc analysis of between-group differences showed significantly greater speed in EXP post-training, but not in pre-training (Table 3).

### 3.3. Standing Performance

While maintaining quiet standing, the differences in sway area between the two groups were nonsignificant pre- and post-training (Table 3). For forward leaning, there was significant group main effect (F(1,30) = 5.461, *p* = 0.026) with greater distance in EXP, but not significant time main effect (F(1,30) = 4.083, *p* = 0.052) or interaction (F(1,30) = 3.928, *p* = 0.057, power = 0.991). For unaffected arm raising speed, the differences between the two groups were nonsignificant pre- and post-training (Table 3).

## 4. Discussion

Improving balance is an important goal in stroke rehabilitation. This study examined the effects of trunk exercises on unstable surfaces on different domains of sitting and standing balance and walking for persons in the subacute stage of stroke. It was found that the experimental training improved the ability to maintain postural stability, lean forward and raise the unaffected arm rapidly in sitting without foot support. These improvements were accompanied by improvements in TIS and 6 m walks. These findings indicated that the experimental training could further improve trunk control, sitting balance and walking ability.

For maintaining balance, the configuration and size of the base of support is a key factor. When seated with feet flat on the ground, not only is the base of support large, the lower legs can also provide passive support [30]. For sitting with foot support, previous studies have used clinical scales, such as the static subscale of TIS, to examine the effects of trunk exercises on static sitting balance of persons in the subacute stage of stroke and attributed a lack of improvement to the measurement ceiling effects [31,32]. This study used a sensitive biomechanical measure and found that postural stability in sitting with foot support also did not improve over time. It should be noted that the participants in this study were able to maintain independent sitting before training. It thus seemed that the ability to maintain sitting postural stability probably might have reached the maximal levels for those who have already gained independent sitting after stroke.

When seated without foot support, the base of support is smaller and trunk control would be primarily responsible for maintaining upright balance. This study found that EXP had a significantly smaller sway area when sitting without foot support after training, suggesting that trunk exercises on unstable surfaces helped to improve trunk control per se. The ability to lean forward in sitting without foot support was also significantly greater for EXP after training, suggesting again that the experimental training improved trunk control per se. This favorable effect, however, did not transfer to forward leaning in sitting with foot support. Loading through the feet has been shown to play an important role in the support of the body during seated forward reach [33], and could possibly mask the contribution of improved trunk control in EXP.

Previous studies examining the effects of trunk exercises on sitting balance in persons in the subacute stage of stroke mostly used TIS as outcome measures and overwhelmingly showed that trunk exercises lead to significantly higher TIS total scores than sham or control training [32,33,34,35]. The findings of this study coincided with the previous findings: EXP had a significantly higher total TIS score than CON. The TIS, however, did not test the ability for the trunk to provide a stable foundation for the performance of the upper limb motion.

In daily living, it is common to perform rapid or forceful movements of the upper limbs in upright posture. These movements not only result in changes in the body configuration and hence change the location of the COM, they will also generate reaction forces acting upon the body [36]. To counteract these imbalancing forces and provide a stable foundation for focal movements, postural adjustments before and during upper limb movements are needed [37,38]. Difficulty in these postural adjustments, e.g., due to stroke or Parkinson’s disease, has been found to be associated with poorer ability in moving the upper limb [10,39]. This study is the first to report that trunk exercises on unstable surfaces significantly improved the ability to raise the unaffected arm rapidly in sitting without foot support, indicating improved trunk control. It should be noted that the grip strength of the unaffected limb did not differ between the two groups, suggesting that faster arm raising speeds after training in EXP were not due to improved unaffected upper limb motor function.

Trunk exercises differed from balance training in that lower limb participation is limited in the former. The carry-over effects of trunk exercise to standing or functional balance tasks for persons in the subacute stage of stroke have been reported previously [32,34,40]. The findings of this study coincided with the previous findings showing that EXP performed significantly better in sitting balance and 6 m walk than CON after training. Considering that the lower limb motor function (i.e., FMLE motor) or plantar sensitivity did not differ significantly between the two groups after training, it seemed likely that the favorable effects on standing balance and walking were related to better trunk control or balance, instead of better lower limb sensorimotor function.

This study was limited in at least two ways. Only people who were able to maintain sitting without support were recruited. It is unclear if the findings could be generalized to people with poorer sitting ability. Trunk alignment might affect the location of the COM relative to the base of support and contribute to balance control, however, it was not monitored in this study.

## 5. Conclusions

Trunk exercises on unstable surfaces could further improve trunk control, the ability to raise the unaffected arm rapidly in sitting, and walking for persons in the subacute stage of stroke. This intervention may be considered to be included in stroke rehabilitation.

## Figures and Tables

**Table 1:** Trunk exercises for the experimental group.

	Progression	Unstable Surface	
		Buttocks	Feet
**Hook-lying**			
1. Abdominal draw-in maneuver	None	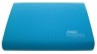	
2. Abdominal muscles isometric contraction	1st		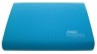
3. Lower trunk rotation	2nd		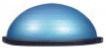
4. Bridging with abdominal draw-in maneuver	3rd	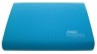	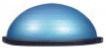
**Sitting Level 1—sitting without foot support**			
1. Pelvic anterior and posterior tilt			
2. Pelvic lateral tilt	1st	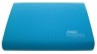	
3. Trunk flexion, extension, rotation			
4. Affected arm lateral reach with Swiss ball under the arm	2nd	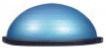	
5. Pelvic rotation			
**Sitting Level 2—sitting on Swiss ball with feet on the ground**			
1. Quiet sitting with chest expansion exercise		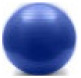	
2. Pelvic anterior and posterior tilt			
3. Pelvic lateral tilt	None		
4. Stepping			
5. Stepping with arm swing			
Note.			
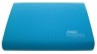	Balance pad		
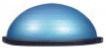	BOSU ball		
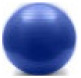	Swiss ball		

**Table 2:** Participant characteristics.

	Control Group (*n* = 17)	Experimental Group (*n* = 18)	*p*
Age (year)	62.4 ± 13.3	60.2 ± 11.7	0.444
Gender (male/female)	9/8	10/8	0.122
Height (cm)	164.1 ± 6.7	166.1 ± 6.7	0.352
Weight (kg)	64.2 ± 10.2	74.6 ± 17.8	0.501
Stroke onset time (weeks)	6.9 ± 2.2	7.0 ± 2.7	0.695
Affected side (left/right)	7 /10	6/12	0.631
Stroke type (infarction/hemorrhage)	6/11	6/12	0.833
**Stroke location (number of participants)**			
Corona radiate	3	2	
Putamen	5	7	
Pons	2	2	
Thalamus	1	1	
Basal ganglion	4	2	
Frontal–temporal lobe	1	0	
Parieto-occipital lobe	0	1	
Corona radiate, basal ganglion, putamen	1	2	
Basal ganglion, thalamus	1	0	
**Modified Ashworth scale (mean/median/mode)**			
Hip adductor	0.1/0/0	0.2/0/0	0.581
Hip flexor	0.0/0/0	0.1/0/0	0.157
Hip extensor	0.1/0/0	0.2/0/0	0.316
Knee extensor	0.1/0/0	0.3/0/0	0.615
Ankle dorsiflexor	0.0/0/0	0.4/0/0	0.212
Ankle plantarflexor	0.3/0/0	0.5/0/0	0.549

**Table 3:** Sensorimotor function, and balance, and walking performance pre- and post-training.

	Control Group		Experimental Group		Pre-Training between-Group Comparisons		Post-Training between-Group Comparisons	
	Pre-Training	Post-Training	Pre-Training	Post-Training	Mean Difference (95%CI)	*p*	Mean Difference (95%CI)	*p*
**Sensorimotor function**								
Unaffected hand grip (kg) ^$^	21.5 ± 12.0	23.5 ± 11.8	21.9 ± 7.9	23.8 ± 8.2		0.386		0.357
Affected plantar sensitivity (log) ^$^	4.8 ± 0.8	4.6 ± 0.8	4.9 ± 1.1	4.9 ± 1.0		0.708		0.708
FMLE motor ^$^	17.4 ± 5.5	18.7 ± 5.3	18.3 ± 6.3	18.6 ± 5.8		0.682		0.97
STREAM ^$^	36.6 ± 16.8	43.2 ± 14.0	40.2 ± 14.5	49.1 ± 13.9		0.369		0.218
TIS total ^#^	14.9 ± 2.7	16.6 ± 1.7	14.6 ± 3.1	17.9 ± 2.3	0.804 (−1.006–2.613)	0.373	−1.458 (−2.814–−0.101)	0.035
6 m walk (s) *^$^	34.4 ± 21.1 (*n* = 12)	36.4 ± 22.5 (*n* = 17)	29.7 ± 14.5 (*n* = 17)	17.3 ± 8.0 (*n* = 18)		0.434		0.012
**Sitting with foot support**								
Static sway area (mm^2^) ^$^	9.7 ± 8.7	11.3 ± 6.9	15.1 ± 18.5	9.5 ± 6.0		0.195		0.195
Forward leaning (mm^2^) ^#^	39.1 ± 17.3	46.1 ± 20.2	55.8 ± 22.5	57.7 ± 18.0	−16.690 (−30.547–−2.832)	0.02	−11.685 (−24.876–1.505)	0.078
Arm raising (degree/sec) ^$^	124.8 ± 54.4	124.4 ± 42.5	132.5 ± 39.6	158.7 ± 41.6		0.732		0.564
**Sitting without foot support**								
Static sway area (mm^2^) ^$^	21.1 ± 18.6	17.4 ± 9.6	15.0 ± 10.5	11.9 ± 5.5		0.684		0.045
Forward leaning (mm) ^^#^	39.7 ± 16.6	41.1 ± 15.7	39.7 ± 13.0	53.4 ± 20.3	0.024 (−10.314–10.362)	0.095	−12.208 (−24.659–−0.242)	0.001
Arm raising (degree/sec) ^#^	132.1 ± 49.7	134.3 ± 41.5	135.8 ± 42.4	157.5 ± 33.3	−3.665 (−35.581–28.252)	0.816	−35.187 (−61.016–−9.359)	0.009
**Standing balance**								
Static sway area (mm^2^) ^$^	675.0 ± 499.2	486.0 ± 413.6	539.8 ± 319.3	385.5 ± 245.8		0.721		0.483
Forward leaning (mm^2^) ^^#^	44.2 ± 17.5	40.4 ± 14.0	50.4 ± 13.1	60.9 ± 17.2	−5.640 (−17.098–5.818)	0.296	−16.076 (−27.338–−4.813)	0.007
Arm raising (degree/sec) ^$^	129.6 ± 58.0	127.7 ± 44.4	130.2 ± 36.3	162.2 ± 42.9		0.935		0.219

FMLE motor: Fugl-Meyer lower extremity motor scale; TIS: trunk impairment scale; STREAM: stroke rehabilitation assessment of movement; CI: confidence interval; *n* = number of participants in the analysis; * participants who were unable to complete the test were excluded from data analysis; ^#^ follow-up independent *t* test after repeated measures ANOVA; ^ group × time interaction nonsignificant; ^$^ *p* values from Mann–Whitney U test.
